# Circulating miR-92b and miR-375 for monitoring the chemoresistance and prognosis of small cell lung cancer

**DOI:** 10.1038/s41598-020-69615-6

**Published:** 2020-07-29

**Authors:** Ming Li, Wulin Shan, Bo Hong, Jinglu Zou, Hong Li, Dandan Han, Yang Zhang, Lailing Li, Dan Li, Wenchu Lin

**Affiliations:** 1 High Magnetic Field Laboratory, Chinese Academy of Sciences, Hefei, 230031 China; 2 University of Science and Technology of China, Hefei, 230036 China; 3 Division of Life Sciences and Medicine, Department of Laboratory Diagnostics, The First Affiliated Hospital of USTC, University of Science and Technology of China, Hefei, 230031 China; 4 Key Laboratory of High Magnetic Field and Ion Beam Physical Biology, Hefei Institutes of Physical Science, Chinese Academy of Sciences, Hefei, 230031 China

**Keywords:** Cancer, Biomarkers, Risk factors

## Abstract

miRNAs have been reported to be stably detectable in plasma and to function as potent biomarkers in multiple cancers. The study aimed to evaluate the expression of candidate circulating miRNAs in patients with small cell lung cancer (SCLC) to identify potential noninvasive biomarkers. The expression of five miRNAs (miR-92b, miR-146a, miR-375, miR-1224, and miR-1246) was significantly upregulated in plasma after chemoresistance induction. Receiver operating characteristic curve (ROC) analysis showed that the area under the curve (AUC) values of miR-92b and miR-375 were 0.766 and 0.791, respectively. The data demonstrated that among the five miRNAs assessed, these two miRNAs had better diagnostic accuracy for monitoring drug resistance. In addition, miR-92b and miR-375 levels were decreased after effective chemotherapy. Furthermore, Kaplan–Meier survival analysis confirmed that high expression of miR-92b and miR-375 was closely related to shorter progression-free survival (PFS) in SCLC patients. In conclusion, these findings indicate that circulating miR-92b and miR-375 might be ideal noninvasive biomarkers for monitoring drug resistance during chemotherapy and evaluating the prognosis of patients with SCLC.

## Introduction

Lung cancer is the deadliest type of cancer worldwide. Small cell lung cancer (SCLC) is a highly malignant cancer type that accounts for 15–20% of lung cancer cases^1,2^. SCLC patients are generally treated with platinum-based chemotherapy alone or in combination with radiotherapy according to tumor stage. Although 60–80% of SCLC patients respond to first-line treatment, the majority inevitably develop chemoresistance and relapse within a relatively short period of time^3^. Identification of biomarkers that can aid in the diagnosis and monitoring of treatment response can have a positive influence on clinical outcomes. Several plasma/serum biomarkers, such as CEA, NSE, and proGRP, have been used for prognosis evaluation under certain circumstances^4,5^. However, these markers are not optimal due to the lack of full sensitivity and specificity. In addition, rare biomarkers have been discovered for monitoring treatment response in SCLC.

miRNAs are a class of conserved small noncoding RNAs (approximately 22 nucleotides in length) that negatively regulate gene expression by binding to target messenger RNAs^6^. Emerging evidence indicates that miRNAs act as crucial regulators in a wide variety of biological processes, such as inflammation, therapy response, differentiation, and migration^7,8^. miRNAs can act as oncogenes by repressing tumor suppressor genes or as tumor suppressors by negatively regulating oncogenes^9^. Since circulating miRNAs are highly stable in plasma/serum and can be detected in smaller quantities in a high-throughput manner, they have great potential to be used as biomarkers in cancer screening and monitoring^10^. Serum levels of miR-21 and miR-92 have been reported to predict recurrence in colon cancer patients^11^. Serum miR-200c expression is correlated with the overall survival of gastric cancer patients^12^. Serum miR-411 has the potential to predict the diagnosis and prognosis of non-small-cell lung cancer^13^. However, circulating miRNAs have been relatively less explored as blood-based biomarkers in SCLC. Hence, this study attempted to explore the relationship between plasma miRNA levels and the malignant behavior of SCLC. The data might contribute to opening novel avenues for monitoring drug resistance and evaluating the prognosis of SCLC.

## Results

### Identification of plasma miRNAs associated with chemotherapy resistance in SCLC

Total RNA was isolated from plasma exosomes of two patients at two stages: newly diagnosed (pre-chemoresistance group) and drug resistant (post-chemoresistance group). miRNA sequencing was performed to assess differential expression between the two groups using next-generation sequencing (NGS) (Supplementary Fig. 1). According to the miRNA profile of these sequencing data and previous studies, 6 candidate miRNAs (miR-92b, miR-146a, miR-199a, miR-375, miR-1224, and miR-1246) were selected for follow-up studies.

**Figure 1:**
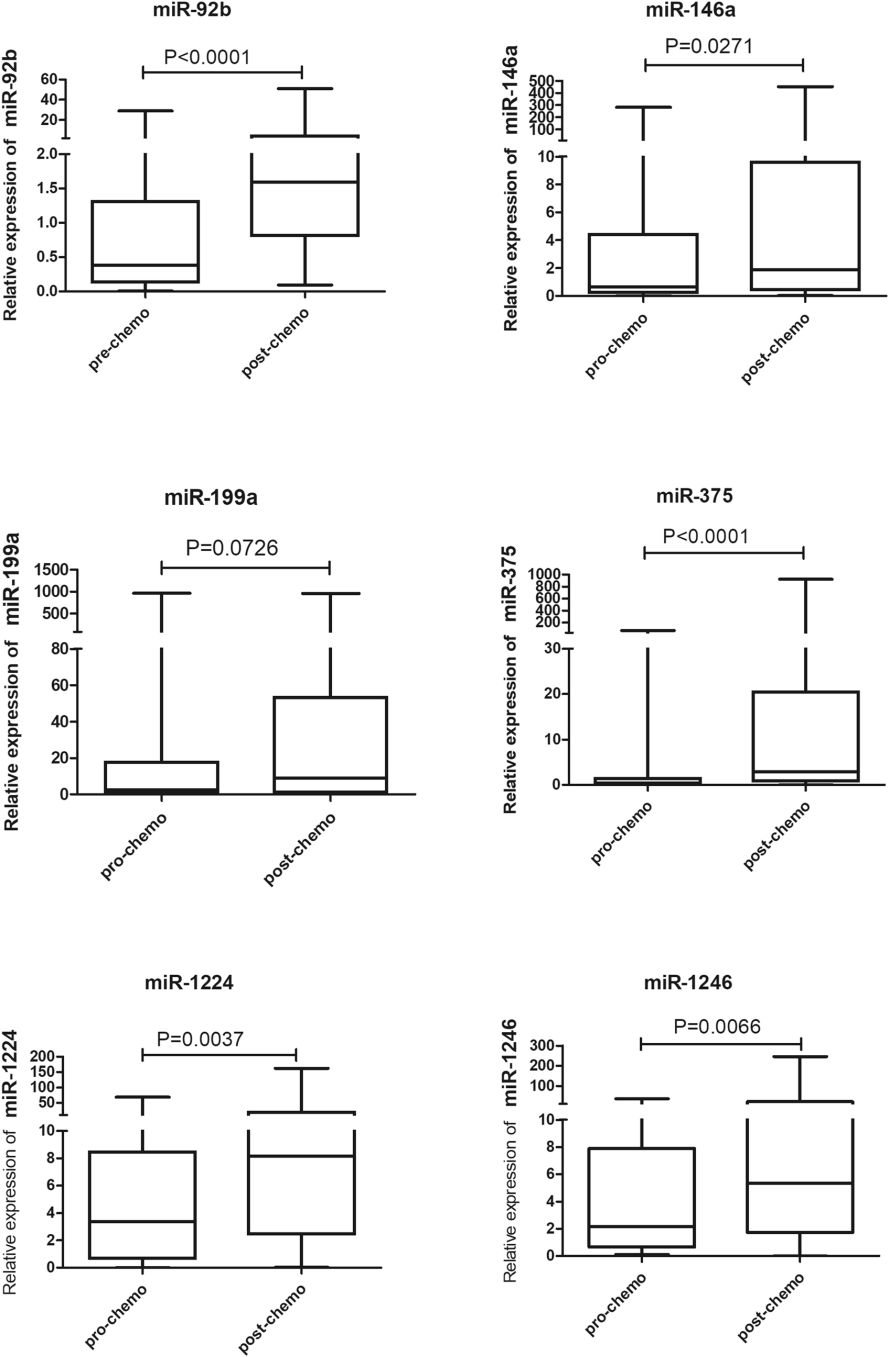
Plasma levels of 6 miRNAs in SCLC patient plasma. The box plots show the plasma levels of miR-92b, miR-146a, miR-199a, miR-375, miR-1224 and miR-1246 at pre- and post-chemoresistance stages.

We first detected the expression of 6 candidate miRNAs in 63 SCLC patients’ plasma at the stages of pre- and post-chemoresistance. Five miRNAs (miR-92b, miR-146a, miR-375, miR-1224, and miR-1246) were significantly higher in the post-chemoresistance group than in the pre-chemoresistance group (*P* < 0.05). Only miR-199a expression did not show a significant change (*P* = 0.0726) (Fig. 1).

### Plasma miRNAs correlate with the clinicopathological features of SCLC

The expression of 5 miRNAs in 38 SCLC patient plasma samples at initial diagnosis was analyzed. The association between miRNA expression and clinicopathological features is presented in Supplementary Table 1. Levels of miR-92b, miR-146a, miR-1224, and miR-1246 were not significantly associated with clinicopathological features (P > 0.05). However, miR-375 expression was positively associated with smoking history (*P* = 0.044) and TNM stage (*P* = 0.003) but was not with age (*P* = 0.305) or sex (*P* = 0.631).

### Clinical value of miRNAs as a factor for monitoring chemotherapy resistance in SCLC

**Figure 2:**
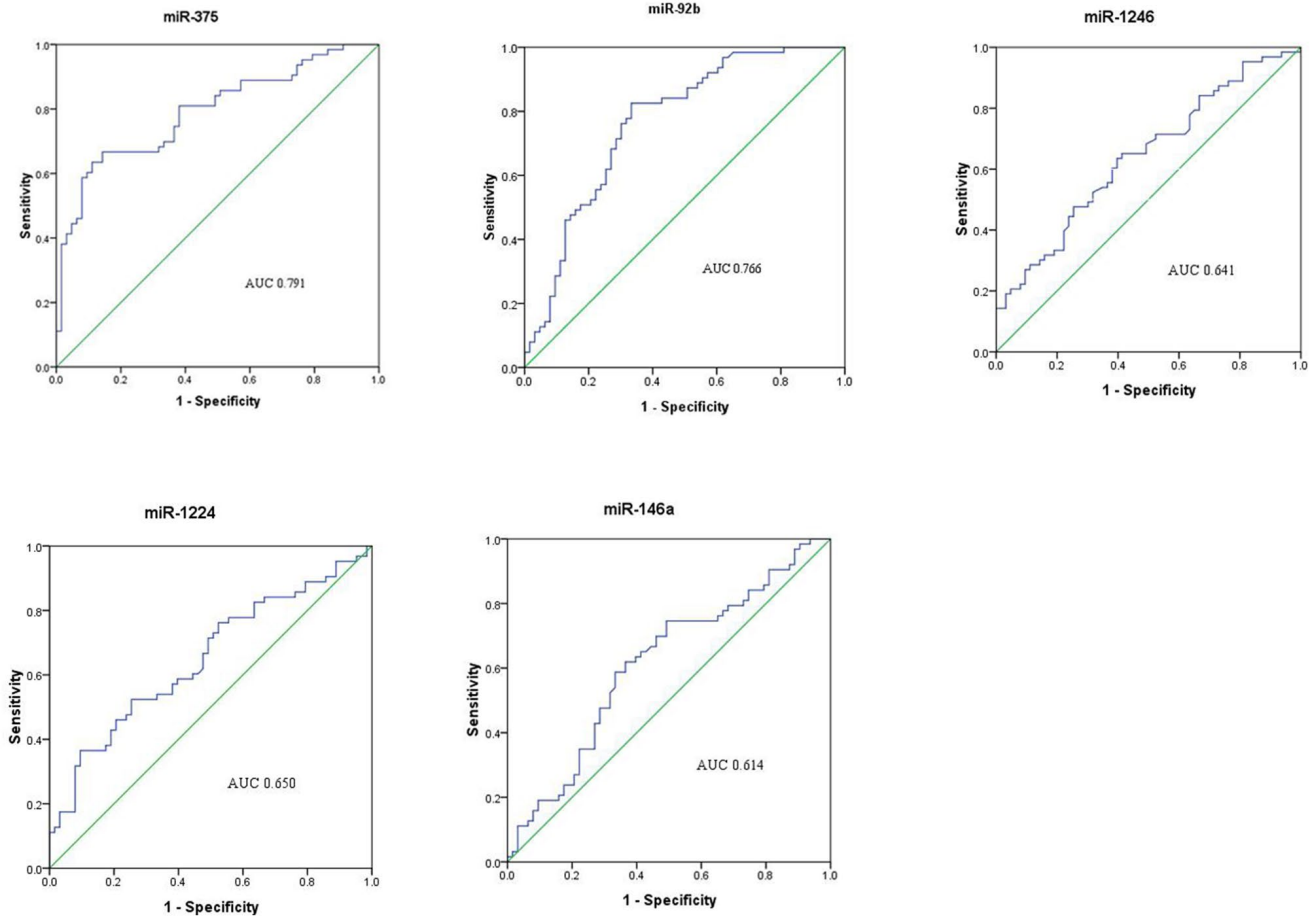
ROC analysis of plasma miRNAs for monitoring chemotherapy resistance in SCLC. AUC estimation for miRNAs in discriminating between pre- and post-chemoresistant patients in the set of 38 SCLC patients.

Receiver operating characteristic curves (ROC) were plotted to determine the diagnostic efficiency of five plasma miRNAs for monitoring chemotherapy response in SCLC. The data showed that miR-375 had the highest area under the curve (AUC) (0.791). MiR-92b had the second-highest AUC (0.785). The AUCs of other miRNAs, including miR-146a, miR-1224, and miR-1246, were lower than 0.700 (Fig. 2). These data indicated that miR-375 and miR-92b are potential biomarkers that can distinguish between post-chemoresistant and pre-chemoresistant SCLC patients.

**Figure 3:**
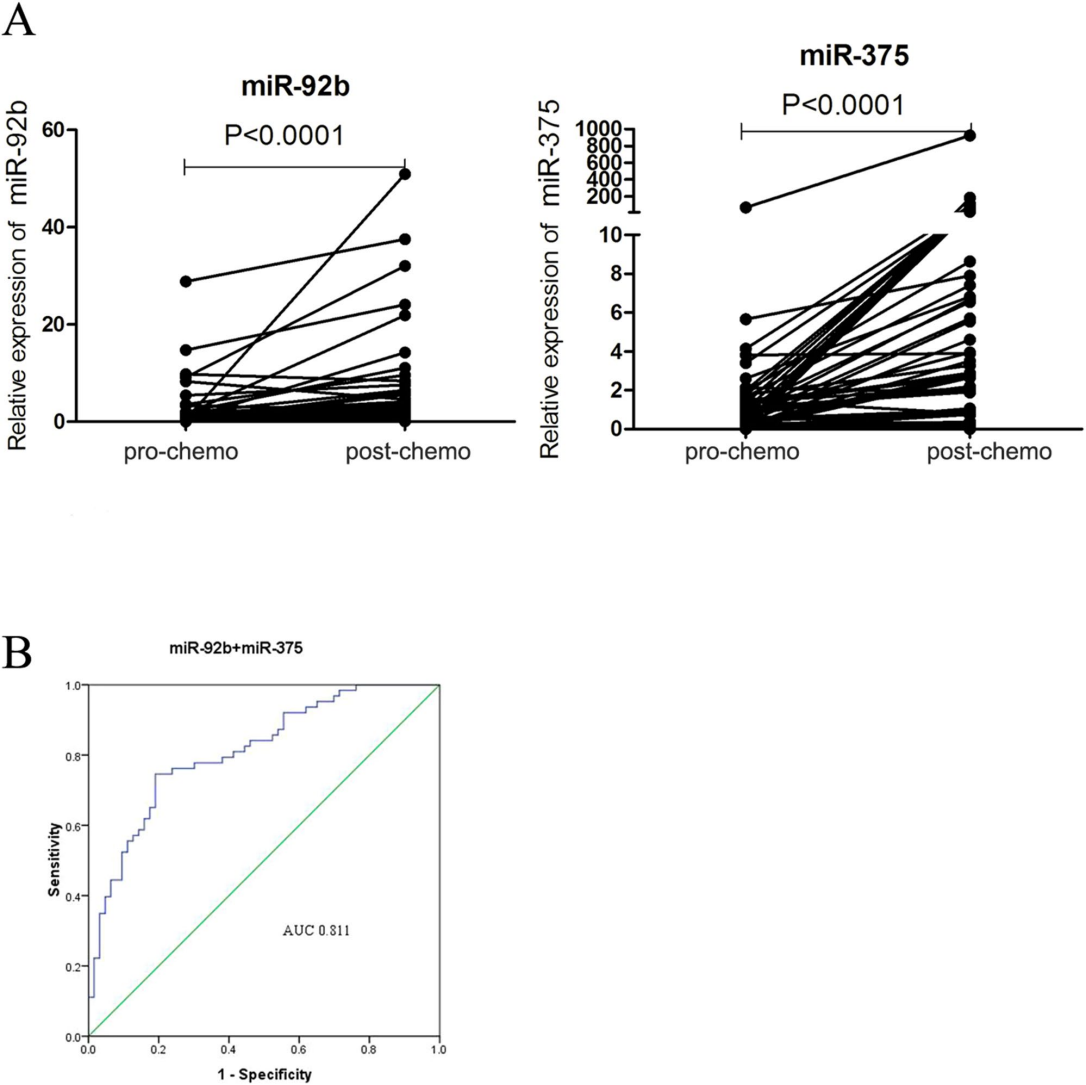
Circulating miRNA levels during treatment and ROC analysis of a two-miRNA panel. (**A**) miR-92b and miR-375 in SCLC patient plasma at the stages of both pre- and post-chemoresistance. (**B**) AUC estimation for miRNAs in discriminating between pre- and post-chemoresistant patients in the set of 38 SCLC patients.

We further calculated the value of two miRNAs (miR-92b and miR-375) for monitoring chemotherapy resistance in SCLC by applying specificity cut-offs. The diagnostic sensitivity of miR-92b and miR-375 was 82.5% and 88.9%, respectively, and the specificity was 66.7% and 61.9%, respectively (Fig. 3A). We also built a panel with two miRNAs (miR-92b and miR-375) to evaluate its predictive value for drug resistance. The diagnostic sensitivity was improved with the panel with two miRNAs, and the AUC was 0.811 (Fig. 3B). Taken together, these data eventually demonstrated that plasma miR-92b and miR-375 could be used for monitoring drug resistance in SCLC.

### Clinical importance of miRNAs as a factor for monitoring chemotherapy response in SCLC

**Figure 4:**
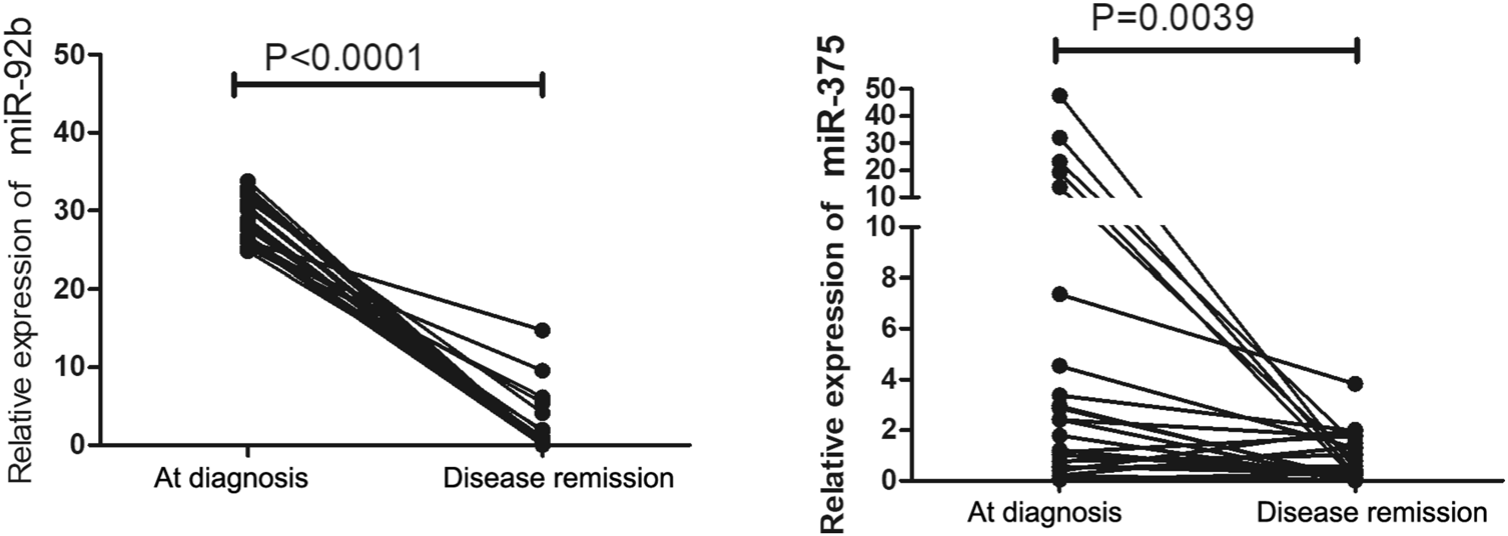
Circulating miRNA levels during chemotherapy. Plasma levels of miR-92b and miR-375 at diagnosis and disease remission.

We further investigated whether two miRNAs (miR-92b and miR-375) could be used as biomarkers for monitoring chemotherapy response in SCLC. Expression of miR-92b and miR-375 in 38 SCLC patients’ plasma at initial diagnosis and disease remission were analyzed by qRT-PCR. The relative levels of miR-92b and miR-375 were significantly lower in the disease remission group than in the initial diagnosis group (P < 0.05) (Fig. 4). These data indicated that these two miRNAs have potential values for monitoring the chemotherapy response in SCLC.

### Plasma miRNAs correlate with a therapeutic effect in SCLC patients

**Figure 5:**
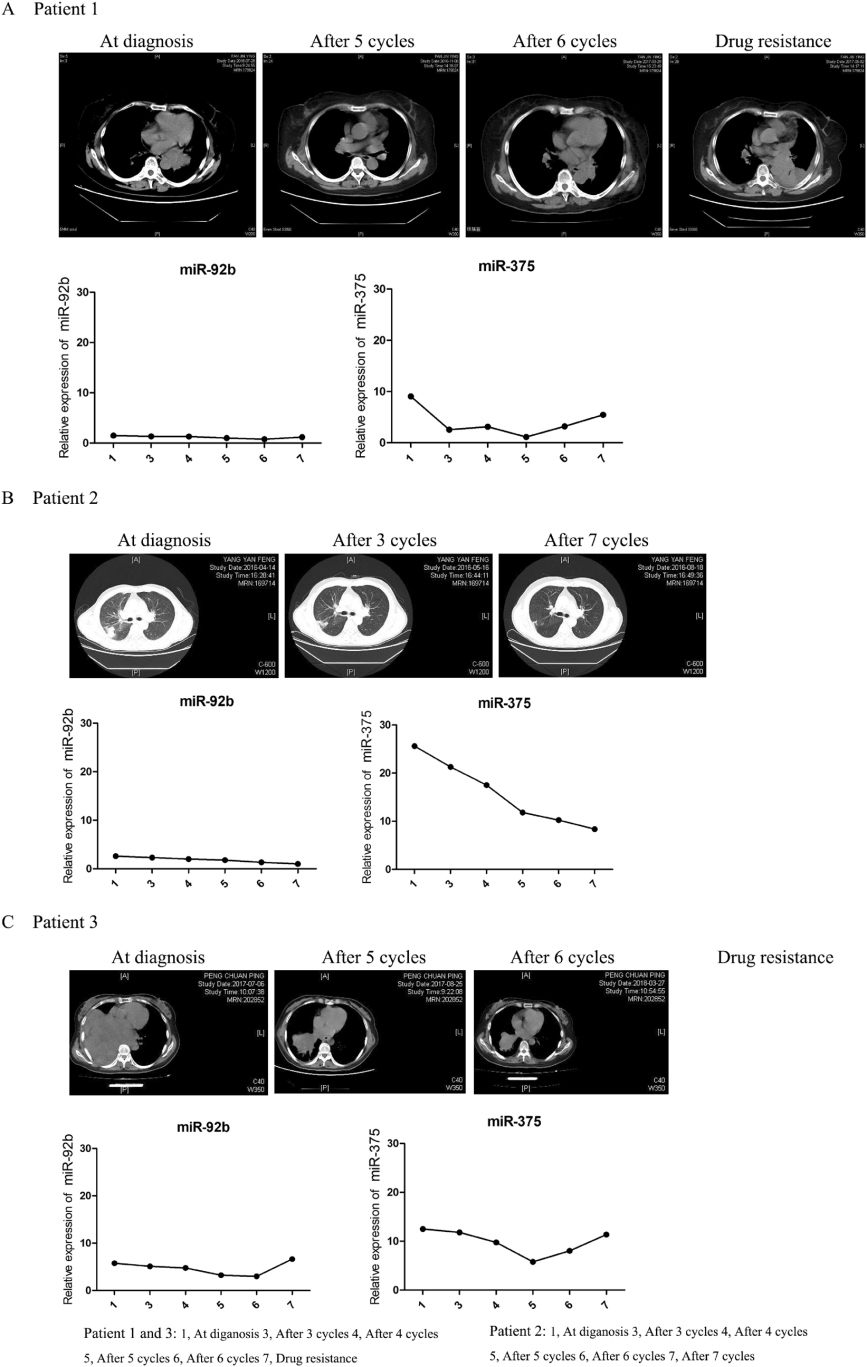
Changes in radiological characteristics and longitudinal analyses of miRNA levels during chemotherapy in three SCLC patients. (**A**) Patient 1. (**B**) Patient 2. (**C**) Patient 3.

Since the expression of two miRNAs (miR-92b and miR-375) appeared to be predictive of therapy response, we performed a longitudinal survey to assess the relationship between radiological characteristics of solid tumors and miRNA levels at several time points during chemotherapy. Patient 2 was in limited-stage at initial diagnosis and still responded to therapy before 7 cycles of chemotherapy. Plasma levels of miR-92b and miR-375 were reduced at the whole time points (Fig. 5B). Patients 1 and 3, who were categorized as the extensive stage at initial diagnosis, responded to therapy before 5 cycles of chemotherapy but later progressed as tumor regrowth after 6 cycles. As shown in Fig. 5A,C, patients 1 and 3 were initially diagnosed with a large solid tumor. After 5 cycles of chemotherapy, tumor size was significantly decreased. However, tumor size started to increase again after 6 cycles of chemotherapy. As with the changes in radiological characteristics, the miR-375 level was steadily decreased before 5 cycles of chemotherapy and increased after 6 cycles of chemotherapy. Although miR-92b expression was also significantly decreased before 5 cycles of chemotherapy, it was not increased at 6 cycles of chemotherapy. These data indicated that plasma miR-92b and miR-375 might be biomarkers for monitoring therapy response in SCLC and suggested that miR-375 may better mirror the response to therapy than miR-92b.

### Clinical importance of miRNAs as prognostic factors for progression-free survival (PFS) in SCLC

**Figure 6:**
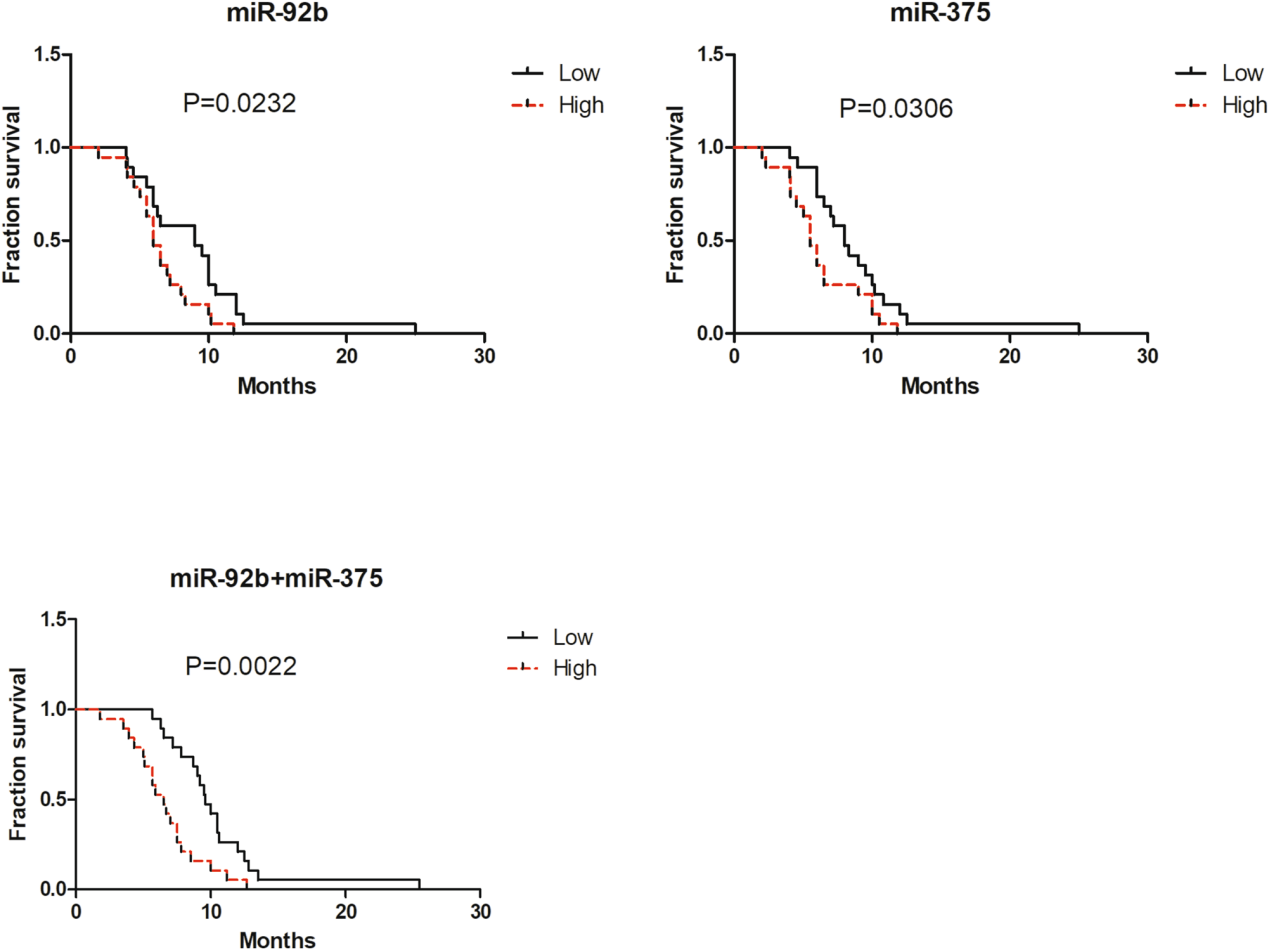
Kaplan–Meier curve for progression-free survival (PFS) for SCLC patients with high levels of miRNAs versus SCLC patients with low levels of miRNAs.

To determine whether two miRNAs (miR-92b and miR-375) were associated with the prognosis of SCLC patients, a prognosis analysis was conducted among 38 SCLC patients by Kaplan–Meier analysis and log-rank test. The PFS of SCLC patients with high miR-92b expression at initial diagnosis was significantly shorter than that of patients with low miR-92b expression (*P* = 0.0232). Similarly, patients with low miR-375 expression had better PFS than those with high miR-375 expression (*P* = 0.0306). Additionally, the combination of these two miRNAs had great value for predicting the PFS of patients (Fig. 6, *P* = 0.0022). These results indicated that miR-92b and miR-375 might be risk factors for progression-free survival in patients with SCLC.

## Discussion

SCLC is characterized by rapid growth and fatal metastasis, ad its clinical prognosis mainly depends on the clinical stage of the tumor. Currently, several plasma/serum biomarkers, including CEA, NSE, and proGRP, have been used for assessing the prognosis of SCLC. However, these markers, to some extent, lack sensitivity and specificity. Circulating miRNAs have attracted much attention since plasma/serum miRNAs are quite suitable for clinical biomarkers for diagnosis and prognosis evaluation^14–16^. Although multiple lines of evidence have shown that miRNAs play critical roles in the proliferation, differentiation, and migration of cancer cells, few studies have assessed the potential value of diagnosis and prognosis evaluation in SCLC. Therefore, we explored the relationship between the expression of plasma miRNAs and chemoresistance and the prognosis of SCLC.

Our study demonstrated that plasma exosomes from SCLC in the initial diagnosis group had a significantly different miRNA expression profile than the drug resistance group by using NGS analysis. The data presented in our study revealed that the expression of 5 miRNAs (miR-92b, miR-146a, miR-375, miR-1224, and miR-1246) was significantly higher after chemotherapy resistance. ROC curve data showed that miR-375 had the highest AUC for monitoring chemoresistance, and miR-92b had moderate diagnostic efficacy. The diagnostic sensitivity of miR-92b and miR-375 was higher than 75%. The combination of the two miRNAs with AUCs of 0.811 improved the diagnostic sensitivity. These results indicated that miR-92b and miR-375 might be informative for assessing chemoresistance in patients with SCLC.

Consistent with our study, several studies reported that miR-92b promoted cancer progression as an oncogene^17,18^. Nakano et al. reported that circulating miR-92b played an essential role in the prediction of posttransplant hepatocellular carcinoma recurrence^19^. The relationship between miR-375 expression and chemoresistance remains controversial. Wang et al. reported that elevated miR-375 expression induced docetaxel resistance in prostate cancer^20^. However, Liu et al. demonstrated that miR-375 overexpression significantly increased the chemosensitivity of breast cancer cells^21^. Yoda et al. reported that cell invasion was promoted by miR-375 overexpression, and high miR-375 expression was correlated with shorter survival time in NSCLC^22^. Zhao et al. indicated that miR-375 was highly expressed in SCLC cells^23^. These data demonstrated that miR-92b and miR-375 might be oncogenes involved in promoting the development and chemoresistance of different types of tumors.

We further investigated whether the expression levels of two miRNAs (miR-92b and miR-375) could be used as biomarkers for monitoring therapy response in SCLC. Our results showed that they were significantly decreased in the disease remission group compared to the chemoresistant group. To further validate our findings, we performed a longitudinal survey in three SCLC patients to assess the relationship between the radiological characteristics of solid tumors and miRNA levels. Our data demonstrated that plasma miR-92b and miR-375 might be biomarkers for monitoring therapy response in SCLC. Finally, we investigated the correlation between the PFS of SCLC patients and the expression of miRNAs. The results indicated that high expression of miR-92b and miR-375 was positively associated with shorter PFS. Overall, these results verified that plasma miR-92b and miR-375 can be used as biomarkers of chemotherapy resistance and prognostic factors for SCLC.

There are some advantages to our research approach. We were able to obtain each matched result from the same patient. Certainly, there were some limitations in this study. First, the lack of NSCLC or age-matched healthy controls was a substantial limitation. Second, our relatively small sample size might limit the generalization of our conclusions and would require further validation in larger cohorts in future studies. In conclusion, our study indicates that plasma miR-92b and miR-375 are candidate biomarkers for monitoring chemoresistance and prognosis in SCLC.

## Materials and methods

### A statement for the study

The Ethics Review Board of the First Affiliated Hospital of the University of Science and Technology of China approved the experiments. Informed consent was obtained from all participants, and all experiments were performed in accordance with relevant guidelines and regulations.

### Patients and clinical samples

This study included 63 SCLC patients from the First Affiliated Hospital of the University of Science and Technology of China whose samples were obtained between January 2015 and October 2018. All samples were collected from consenting individuals according to the protocols approved by the Ethics Review Board of the First Affiliated Hospital of the University of Science and Technology of China. None of the patients had undergone treatment before enrollment. All participants underwent strict imaging and physical examinations. Demographic characteristics and medical and smoking histories were collected. All of the enrolled patients were diagnosed by pathology. Of the 63 SCLC patients, 53 were men (82.5%), and 10 were women (17.5%). The median age was 64 years (range 46–85 years). Forty (63.5%) patients were nonsmokers, and the other 23 (26.5%) were current or ex-smokers. Twelve (19.0%) patients had limited-stage disease, and 51 (81.0%) had extensive-stage disease at initial diagnosis. At least six cycles of chemotherapy were administered to all 63 patients.

Plasma samples were selected retrospectively at the time of analysis according to the following requirements: (1) plasma was collected at three stages: diagnosis, pre-chemoresistance (when patients responded to chemotherapy) and post-chemoresistance (when patients had recurrence or progressive disease during chemotherapy). (2) A sufficient volume of plasma was available for RNA isolation.

### miRNA isolation and qRT-PCR assay

Peripheral blood samples (2 ml) were obtained at each stage from patients and placed in a plasma separator tube. Separation of the plasma was accomplished by centrifugation at 3,500 r.p.m for 10 min at room temperature. The supernatant plasma was recovered and stored at 80 ℃ until analysis.

Total RNA was isolated from 200 μl plasma samples using the miRNeasy Mini Kit (Qiagen, Germany) according to the manufacturer’s protocol. A total of 25 fmol synthetic *C. elegans* miRNA (cel-miR-39-3p, Qiagen) was introduced after the addition of denaturing solution to each sample to monitor technical variations in RNA extraction, as described previously. A260/A280 ratio was used to quantify the extracted RNA by Qubit 4.0 (Life technologies, USA). qRT-PCR was run on a Roche LC480 system (Roche, Germany), and the reaction mixtures were incubated at 95 °C for 10 min, followed by 40 cycles of 95 °C for 15 s and 60 °C for 1 min. Relative Ct values were normalized using the cel-miR-39-3p Ct value. The relative quantification of miRNA expression was calculated with the 2^−ΔΔCt^ method. All real-time analyses performed in triplicate for all samples.

### Statistical analysis

Statistical analyses were carried out using SPSS 19.0 software. The Mann–Whitney U test was performed to compare the levels of plasma miRNAs at the stages of diagnosis, disease remission, and drug resistance of SCLC patients. Associations between clinicopathological parameters and the expression of plasma miRNAs were evaluated using chi-square tests. Survival curves were constructed with the Kaplan–Meier method and compared by log-rank tests. *P* values < 0.05 were considered statistically significant.

### Informed consent

All enrolled patients signed informed consent forms.

## Supplementary information

Supplementary information.
