# Supplementary material for: Circulating miR-92b and miR-375 for monitoring the chemoresistance and prognosis of small cell lung cancer
Source: Sci Rep. 2020 Jul 29;10:12705. doi: 10.1038/s41598-020-69615-6 (PMC7391689; doi:10.1038/s41598-020-69615-6)
Supplement: Supplementary file 1 — Supplementary information. [file 41598_2020_69615_MOESM1_ESM.doc]

| **Supplementary Figure 1.** Heatmap of miRNAs between the pre-chemoresistance and post-chemoresistance groups.  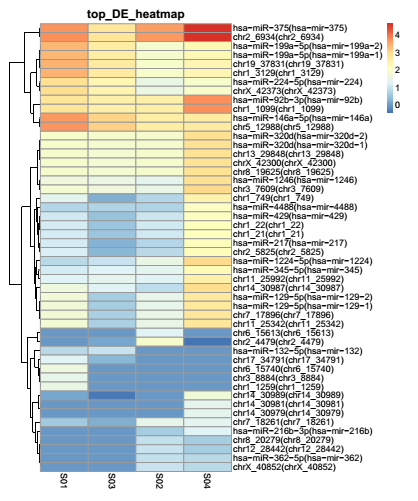  **Supplementary Table 1.**  Association of miRNAs with clinicopathological features of SCLC patients at diagnosis | | | | | | | | | | | | | | | |
| --- | --- | --- | --- | --- | --- | --- | --- | --- | --- | --- | --- | --- | --- | --- | --- |
| Features | miR-92b expression | | P | miR-146a expression | | P | miR-375 expression | | P | miR-1224 expression | | *P* | miR-1246 expression | | *P* |
|  | Low | **High** |  | Low | **High** |  | Low | **High** |  | Low | **High** |  | Low | **High** |  |
| **Age (years)** |  |  |  |  |  |  |  |  |  |  |  |  |  |  |  |
| <=60 | 5 | **8** | **0.305** | 5 | **8** | **0.305** | 8 | **5** | **0.305** | 7 | **6** | **0.732** | 6 | 7 | 0.732 |
| >60 | 14 | **11** |  | 14 | **11** |  | 11 | **14** |  | 12 | **13** |  | 13 | 12 |  |
| **Gender** |  |  |  |  |  |  |  |  |  |  |  |  |  |  |  |
| Female | 1 | **4** | **0.15** | 3 | **2** | **0.631** | 3 | **2** | **0.631** | 3 | **2** | **0.631** | 1 | 4 | 0.15 |
| Male | 18 | **15** |  | 16 | **17** |  | 16 | **17** |  | 16 | **17** |  | 18 | 15 |  |
| **Smoking history** |  |  |  |  |  |  |  |  |  |  |  |  |  |  |  |
| No | 13 | **11** | **0.501** | 13 | **11** | **0.501** | 15 | **9** | **0.044** | 11 | **13** | **0.501** | 11 | 13 | 0.501 |
| Yes | 6 | **8** |  | 6 | **8** |  | 4 | **10** |  | 8 | **6** |  | 8 | 6 |  |
| **TNM stage** |  |  |  |  |  |  |  |  |  |  |  |  |  |  |  |
| I–II | 7 | **2** | **0.056** | 7 | **2** | **0.056** | 9 | **1** | **0.003** | 7 | **2** | **0.056** | 5 | 4 | 0.703 |
| II–IV | 12 | **17** |  | 12 | **17** |  | 10 | **18** |  | 12 | **17** |  | 14 | 15 |  |
